# A novel nutritional score based on serum triglyceride and protein levels predicts outcomes of intrahepatic cholangiocarcinoma after curative hepatectomy: A multi-center study of 631 patients

**DOI:** 10.3389/fnut.2022.964591

**Published:** 2022-09-21

**Authors:** Yunshi Cai, Shuai Xue, Jiaxin Li, Heng Xiao, Tian Lan, Hong Wu

**Affiliations:** ^1^State Key Laboratory of Biotherapy and Cancer Center, Department of Liver Surgery and Liver Transplantation, West China Hospital, Sichuan University and Collaborative Innovation Center of Biotherapy, Chengdu, China; ^2^Department of Hepatobiliary Surgery and Liver Transplantation, The First Affiliated Hospital of Chongqing Medical University, Chongqing, China

**Keywords:** serum triglyceride, intrahepatic cholangiocarcinoma, albumin, globulin, nutritional score

## Abstract

**Background:**

High serum triglyceride (STG) level is a well-established pathogenic factor for cardiovascular diseases and is associated with the risk of various malignancies. Nevertheless, the role of STG level in intrahepatic cholangiocarcinoma (ICC) remains uncertain.

**Methods:**

A total of 631 ICC patients treated with curative hepatectomy in two centers (517 in the discovery set and 114 in the validation set) were retrospectively analyzed. Kaplan–Meier survival analysis was used to assess the outcomes of the patients with different STG levels. Time-dependent receiver operating characteristic (ROC) analysis was conducted to compare the prognostic value of STG with other established indexes. The Triglyceride-Albumin-Globulin (TAG) grade was introduced and evaluated using the time-dependent area under curves (AUC) analysis and decision curve analysis (DCA).

**Results:**

Patients with increased STG levels and decreased albumin-globulin score (AGS) were correlated with improved overall survival (OS) and recurrence-free survival (RFS). STG level ≥ 1 mmol/L was an independent protective factor for surgically treated ICC patients. The predictive value of the TAG grade was superior to the STG or the AGS alone. In decision curve analysis, the net benefits of the TAG grade in the discovery and validation set were higher than STG and AGS.

**Conclusion:**

The current study presented strong evidence that ICC patients with higher preoperative STG levels had preferred long-term surgical outcomes. The novel nutritional score based on serum triglyceride, albumin and globulin levels was inextricably linked to the prognosis of the surgically treated ICC patients. Evaluation of the TAG grade before curative hepatectomy may be beneficial for risk stratification and clinical decision support.

## Introduction

Liver cancer remains the fifth leading cause of all-cause mortality and the second most common cause of cancer-related death in China (1, 2), which leads to 37 per 10000 people of newly diagnosed cases annually (2). ICC represents a major subtype of biliary tract cancer located within the liver parenchyma (3), accounting for nearly 20% of primary liver cancers (4), of which the incidence and mortality are increasing rapidly in recent years (5). ICC is often diagnosed at an advanced stage with limited treatment options available; surgical resection remains one of the major treatment modalities (6). Recurrence is frequent after liver resection, resulting in a poor prognosis with less than 40% of surgically treated ICC patients on survive more than 5 years (7). Therefore, prognostic indicators are required to evaluate the outcomes of the surgical candidates and carry out early interventions, thus improving postoperative survival.

The population with ALD and NAFLD in China increased sharply (8, 9), as a result, the incidence and mortality of liver cancer started to soar since 2015 after a short duration of decline in the first decade of the 21st century (10). Previous studies had demonstrated that the metabolic syndrome (obesity, dyslipidemia, hypertension, and impaired fasting glucose/diabetes mellitus) was associated with increased risk of ICC (odds ratio = 1.56, *P* < 0.0001), whereas treatment with metformin was significantly related to a reduction of ICC risk (11, 12). High STG level, as an essential component of metabolic syndrome, was positively associated with the risk of multiple malignancies (lung, rectal, thyroid, renal, and gynecological cancers) and was inversely associated with the risk of non-Hodgkin’s lymphoma and prostate cancer (13); however, the correlation between STG concentration and the prognosis of ICC patients have been inconclusive (14, 15).

As crucial components of human serum proteins, albumin (ALB) and globulin (GLB) levels were widely used in nutritional assessment. Previous studies have presented that elevated levels of serum ALB increased the risks of esophageal and cervical cancers (16, 17). GLBs, a group of proteins that binds estrogen, dihydrotestosterone and testosterone, play an essential role in the inflammatory immune response (18). Moreover, the high pretreatment ALB/GLB ratio has been proved to be associated with increased 5-year mortality and recurrence in different human cancers, including breast and gastric cancers (19). Similarly, another model based on serum ALB and GLB level, the AGS, has been applied in predicting the prognosis of non-small-cell lung cancer (20). As well, our recent study showed a positive correlation between the AGS and the long-term outcomes of ICC patients (21).

In the current study, we aimed to determine the correlation between STG and the long-term survival of surgically treated ICC patients. In addition, a novel nutritional score based on the STG concentration and the AGS (Triglyceride-Albumin-Globulin, TAG) was generated; we hoped to evaluate the clinical efficacy of the TAG grade in the prognosis of ICC patients after curative surgery.

## Materials and methods

### Study population

A total of 631 patients ICC patients received curative resection at two medical centers, West China Hospital of Sichuan University and the First Affiliated Hospital of Chongqing Medical University, were sequentially enrolled. 517 patients underwent surgery at the West China Hospital during December 2008 and December 2017 were included as discovery set, 114 patients underwent surgery at the First Affiliated Hospital of Chongqing Medical University between May 2010 and December 2015 were included as the validation set to verify the efficiency of the novel scoring system. The inclusion criteria were as follow: (1) histologically diagnosed ICC; (2) underwent liver resection with curative intent initially; (3) no lipid-lowering treatment within 2 weeks prior to the operation; (4) serum TG measured in fasting status; exclusion criteria: (1) patients underwent preoperative RFA, TACE, radiation treatment, targeted therapy, or other anti-cancer treatment; (2) extrahepatic metastasis before the operation; (3) positive resection margin; (4) ruptured tumor; (5) patients with incomplete clinical and histological data or lost to follow-up. Written informed consents were obtained from all participants or their entrusted agents. This study was approved by the ethics committee of West China Hospital of Sichuan University (Approval number: 20211506) and the First Affiliated Hospital of Chongqing Medical University (Approval number: 2021-288), following the guidelines of the 1975 Declaration of Helsinki.

### Data collection and follow-up

All the clinical and histopathological data were accessed from the electronic medical record system. Preoperative information was collected as follows: platelet counts; bilirubin, albumin (ALB), and globulin (GLB) levels; triglyceride (TG), cholesterol (CHOL), and low-density lipoprotein (LDL-C), ALT, and AST levels; HBV and HCV viral loads; carbohydrate antigen 19-9 (CA19-9) level; furthermore, the height and weight of the patients were obtained to calculate the body mass index (BMI) as weight divided by height squared (kg/m^2^). The normal range of the TG was 0.29–1.83 mmol/L according to the determination kit’s instruction. The optimal cut-off values of the TG, CHOL and LDL-C were determined as 1, 3.8, and 2.6 mmol/L, respectively (Supplementary Figure 1). As we previously reported, the cut-off values of the ALB and GLB were 41.7 and 28.6 g/L, respectively (21); the AGS was characterized as following criteria: patients with both normal values of the ALB (>41.7 g/L) and GLB (≤28.6 g/L) were defined as AGS 0, patients with both decreased ALB level (≤41.7 g/L) and increased GLB level (>28.6 g/L) were defined as AGS 2, those with single abnormal value were defined as AGS 1. Fibrosis-4 index (FIB-4) was calculated by $\left(A\,L\,T\,\left(U/L\right)\times a\,g\,e\,\left(y\,e\,a\,r\right)\right)/\left(p\,l\,a\,t\,e\,l\,e\,t\,\left(10^{9}/L\right)\times \sqrt{A\,S\,T\,\left(U/L\right)}\right)$ (22); albumin-bilirubin index (ALBI) was calculated from the following formula: (*log*_10_*bilirubin*(*mol*/*L*)×0.66)−(*albumin*(*g*/*L*)×0.085) (23). SMI was calculated as described in our previous study (21). The cut-off values were identified by using the X-tile software (24). Clinical and histological characteristics, including the presence of liver cirrhosis, ascites and hepatolithiasis, numbers and diameters of the tumor nodules, differentiation, lymph node positivity, microvascular invasion (MVI), vascular and perineural invasion, were also attained. MVI was defined as the presence of tumor cells in vessels or in vascular space lined by the epithelial cells under the microscope. Vascular invasion was defined as large vessels invasion identified by imaging modalities or gross examinations. Positivity of No. 16 lymph node was regarded as extrahepatic metastasis and radical resection was not considered. The tumor-node-metastasis (TNM) stages were classified conforming to the 8th American Joint Committee on Cancer (AJCC) Staging Manual (25). Patients who received curative resections were followed-up every month within 1 year, then every 3 months within the first 2 years, and then every half year thenceforth, serum tumor markers and imaging methods (contrast-enhanced ultrasound or CT scan) were used in the surveillance of tumor recurrence. The overall survival (OS) was calculated from the date of curative hepatectomy to the date of death or the last follow-up (for those alive). The recurrence-free survival (RFS) was calculated from the date of curative hepatectomy to the detection of recurrence or the date of the last follow-up (for those without tumor relapse).

### Statistical analysis

The software of EmpowerStats^1^ and R^2^ (v4.0.5) was used for statistical analysis; data were presented as mean ± standard deviation (SD), median (interquartile range) or proportion. Comparison of categorical and continuous variables between groups was performed with Student’s *t*-test, Pearson’s *x*^2^ test and Analysis of variance (ANOVA). Non-parametric Mann–Whitney *U* test and Kruskal–Wallis test were used to analyze the data with the abnormal distribution. The ideal cut-off values of TG, CHOL, LDL-C, ALB, GLB, FIB-4, and ALBI were identified by using the software of X-tile^3^. The discriminatory ability of the indexes was assessed by the time-dependent area under receiver operating characteristic (AUROC) analysis via the “survivalROC” package in R. Comparison between ROC curves were performed by using DeLong’s test via the “pROC” package in R. Kaplan–Meier curves were depicted according to the optimal cut-off values, and their differences between groups were determined by comparing the cumulative survival of the included ICC patients using the log-rank test. Cox proportional hazards regression model was used to identify potential prognostic factors for OS and RFS; clinical and histological parameters with *P* < 0.2 in the univariate model were integrated into the multivariate model. Comparisons between the complex and straightforward models were conducted through decision curve analysis (DCA) via the “rmda” package in R. *P* < 0.05 was considered statistically significant.

## Results

### Baseline characteristics of the patients

Five hundred seventeen patients [251 (48.5%) male, mean (*SD*) age, 57.2 (10.7) years] in West China Hospital and 114 patients [49 (43.0%) male, mean (*SD*) age, 58.3 (11.2) years] in the First Affiliated Hospital of Chongqing Medical University were finally included into the discovery set and validation set, respectively. The BMI of the patients was lower in the discovery set (24.8 ± 6.2 kg/m^2^) than those in the validation set (25.3 ± 5.8 kg/m^2^). Liver cirrhosis was observed in 145 (28.0%) patients in the discovery set and 16 (14.0%) patients in the validation set. All patients were classified into Child-Pugh grade A, among which 439 (84.9%) patients in the discovery set and 86 (75.4%) patients in the validation set were Child-Pugh score 5. Multiple tumor nodules were detected in nearly 30% of the patients in both sets (156 (30.2%) in the discovery set and 32 (28.1%) in the validation set). Less than half of the patients in both sets were with tumor nodules greater than 5 cm (225 (43.5%) in the discovery set and 46 (40.4%) in the validation set). 87 (16.8%) of the patients in the discovery set and 21 (18.4%) of the patients in the validation set were with intrahepatic calculus. Positive lymph nodes were revealed in 127 (24.6%) patients in the discovery set and 31 (27.2%) patients in the validation set, respectively. The average TG levels were 1.3 ± 0.6 mmol/L in the discovery set and 1.3 ± 0.8 mmol/L in the validation set. The average ALB and GLB levels were slightly higher in the discovery set (ALB: 42.5 ± 4.7 g/L, GLB: 29.1 ± 5.4 g/L) than those in the validation set (ALB: 38.6 ± 6.9 g/L, GLB: 26.9 ± 5.2 g/L). Additionally, more than half of the patients [361 (69.8%) in the discovery set and 71 (62.3%) in the validation set] were stratified into TNM stage III. Patients’ characteristics at baseline were summarized in Table 1.

**TABLE 1:** Baseline characteristics of patients.

Variables	Discovery set (*n* = 517)	Validation set (*n* = 114)	*P*-value
Age, year, mean ± *SD*	57.2 ± 10.7	58.3 ± 11.2	0.327
**Gender, *n* (%)**			0.281
Male	251 (48.5%)	49 (43.0%)	
Female	266 (51.5%)	65 (57.0%)	
BMI kg/m^2^, mean ± *SD*	24.8 ± 6.2	25.3 ± 5.8	0.436
Fasting glucose (mmol/L), mean ± *SD*	5.9 ± 2.1	5.6 ± 2.3	0.249
Cirrhosis, *n* (%)	145 (28.0%)	16 (14.0%)	0.007
Ascites, *n* (%)	48 (9.3%)	55 (48.2%)	<0.0001
**Child score, n (%)**			0.014
5	439 (84.9%)	86 (75.4%)	
6	78 (15.1%)	28 (24.6%)	
Multiple tumors, *n* (%)	156 (30.2%)	32 (28.1%)	0.709
**Tumor size, *n* (%)**			0.64
≥5 cm	225 (43.5%)	46 (40.4%)	
<5 cm	292 (56.5%)	68 (59.6%)	
Tumor differentiation, poor, *n* (%)	325 (66.7%)	84 (73.7%)	0.194
Hepatolithiasis, *n* (%)	87 (16.8%)	21 (18.4%)	0.709
Microvascular invasion, *n* (%)	52 (10.1%)	70 (61.4%)	<0.0001
Vascular invasion, *n* (%)	120 (23.2%)	71 (62.3%)	<0.0001
Node positivity, *n* (%)	127 (24.6%)	31 (27.2%)	0.611
Biliary invasion, *n* (%)	52 (10.1%)	58 (50.9%)	<0.0001
Perineural invasion, *n* (%)	75 (14.5%)	25 (21.9%)	0.071
Liver capsule invasion, *n* (%)	319 (61.7%)	62 (54.4%)	0.363
CA19-9, ≥22 U/mL, *n* (%)	113 (21.9%)	85 (74.6%)	<0.0001
HBsAg (positive), *n* (%)	150 (29.1%)	21 (18.4%)	0.049
HCV, *n* (%)	4 (0.8%)	1 (0.9%)	0.91
TNM stage, III, *n* (%)	361 (69.8%)	71 (62.3%)	0.378
TG, mmol/L, mean ± *SD*	1.3 ± 0.6	1.3 ± 0.8	0.426
ALB, g/L, mean ± *SD*	42.5 ± 4.7	38.6 ± 6.9	<0.0001
GLB, g/L, mean ± *SD*	29.1 ± 5.4	26.9 ± 5.2	0.005
SMI, male, mean ± *SD*	41.4 ± 6.8	42.6 ± 7.4	0.356
SMI, female, mean ± *SD*	36.5 ± 7.1	35.7 ± 7.8	0.562
Overall survival, month, median (interquartile range)	17.8 (9.8–33.6)	25.1 (9.6–58.8)	0.006

CA19-9, cancer antigen 19-9; HBsAg, hepatitis B surface antigen; HCV, hepatitis C virus; TG, triglyceride; ALB, albumin; GLB, globulin; SMI, skeletal muscle index.

### Serum triglyceride level was an independent protective factor for intrahepatic cholangiocarcinoma

Several preoperative indexes related to serum lipid levels (TG, CHOL, and LDL-C) were compared, among the three indexes, only the serum TG level presented the discriminative ability for surgically treated ICC patients (Supplementary Figure 1); likewise, the upper limit of normal (ULN, 1.83 mmol/L) of TG was evaluated, no significant difference in the OS and RFS between the patients with and without hyperlipidemia (Supplementary Figure 2).

**FIGURE 1:**
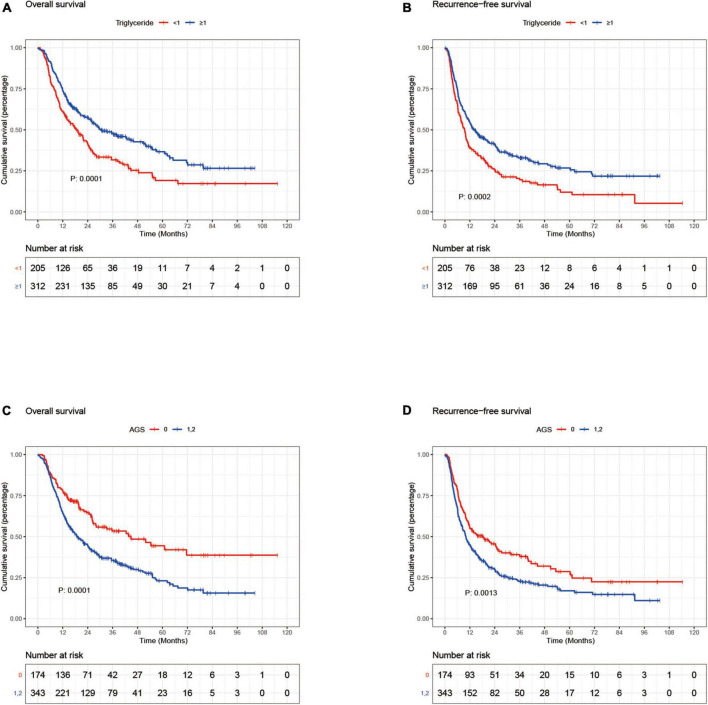
Kaplan–Meier curves for overall survival and recurrence-free survival stratified by triglyceride **(A,B)** and AGS **(C,D)** in the discovery set; AGS, albumin-globulin score.

**FIGURE 2:**
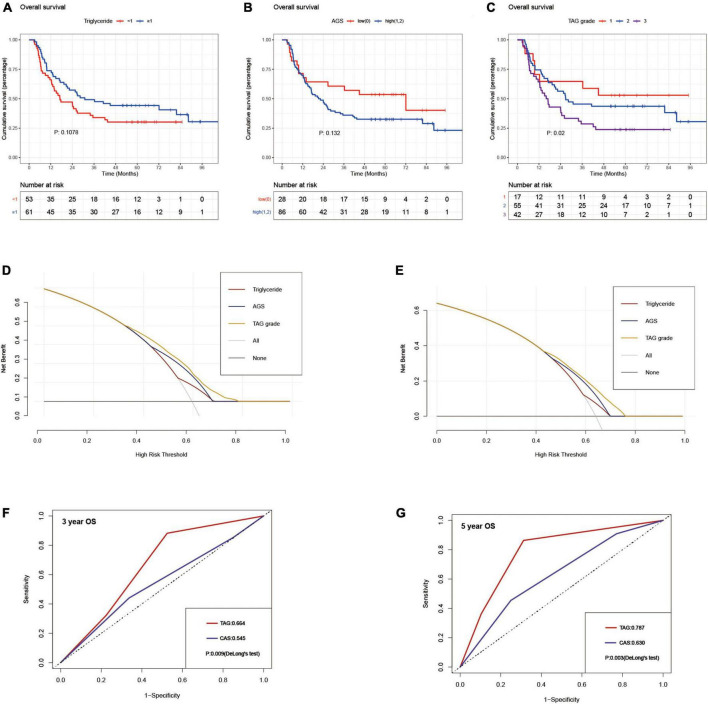
Kaplan–Meier curves for overall survival stratified by triglyceride **(A)**, AGS **(B)**, and TAG grade **(C)** in the validation set; decision curve analyses for overall survival of triglyceride, AGS and TAG grade in the discovery set **(D)** and validation set **(E)**; comparison of area under receiver operator characteristic curves for TAG and CAS grade in predicting 3-year **(F)** and 5-year **(G)** overall survival in the validation set using DeLong’s test; AGS, albumin-globulin score; TAG grade, triglyceride-albumin-globulin grade; CAS grade, combination of albumin-globulin score and skeletal muscle index.

Therefore, 1 mmol/L was determined as the optimal cut-off value for serum TG level; 312 patients were with higher TG levels (≥1 mmol/L) and 205 patients were with lower levels (<1 mmol/L) in the discovery set, the correlations of characteristics with the serum triglyceride levels in the discovery and validation sets were demonstrated in Table 2 and Supplementary Table 1, respectively. Kaplan–Meier analysis suggested that the patients with higher TG levels had better OS and RFS than those with lower TG levels (median OS: 27.1 months vs. 21.5 months) (Figures 1A,B), a similar difference was further found in the validation set (Figure 2A). Models represented the preoperative hepatic function including the FIB-4 and ALBI score were also calculated; the discriminative capability of these two models and TG level to 1-, 3-, and 5-year OS and RFS were compared by using ROC; the AUC of TG were superior to that of FIB-4 and ALBI (Supplementary Figure 3). Consistently, multivariate analyses using the Cox proportional hazard model revealed that increased serum TG level was an independent protective factor for both the OS and RFS (OS: Hazard ratio, 0.66, *P* = 0.0014; RFS: Hazard ratio, 0.69, *P* = 0.0012) (Table 3).

**TABLE 2:** Comparison of characteristics with the different serum triglyceride levels and AGS grades of 517 ICC patients treated with surgical resection in the discovery set.

Characteristics	STG (mmol/L)			AGS		
	<1 (*n* = 205)	≥1 (*n* = 312)	*P*-value	Low (0) (*n* = 174)	High (1, 2) (*n* = 343)	*P*-value
Age, mean ± *SD*	55.6 ± 10.9	58.3 ± 10.3	0.09	56.5 ± 11.0	57.6 ± 10.5	0.339
Gender, male, *n* (%)	103 (50.2%)	148 (47.4%)	0.532	88 (50.6%)	163 (47.5%)	0.512
BMI kg/m^2^, mean ± *SD*	24.6 ± 3.4	26.4 ± 4.9	0.663	26.53 ± 4.2	25.74 ± 5.6	0.781
Cirrhosis, *n* (%)	65 (31.7%)	80 (25.6%)	0.133	50 (28.7%)	95 (27.7%)	0.804
Ascites, *n* (%)	21 (10.2%)	27 (8.7%)	0.542	12 (6.9%)	36 (10.5%)	0.183
Multiple tumors, *n* (%)	60 (29.3%)	96 (30.8%)	0.716	47 (27.0%)	109 (31.8%)	0.265
Tumor size (<5 cm), *n* (%)	115 (56.1%)	177 (56.7%)	0.887	77 (44.3%)	148 (43.1%)	0.811
Hepatolithiasis, *n* (%)	38 (18.5%)	49 (15.7%)	0.4	27 (15.5%)	60 (17.5%)	0.57
Microvascular invasion, *n* (%)	26 (12.7%)	26 (8.3%)	0.108	17 (9.8%)	35 (10.2%)	0.877
Vascular invasion, *n* (%)	49 (23.9%)	71 (22.8%)	0.763	37 (21.3%)	83 (24.2%)	0.455
Node positivity, *n* (%)	61 (29.8%)	66 (21.2%)	0.046	34 (19.5%)	93 (27.1%)	0.059
Biliary invasion, *n* (%)	18 (8.8%)	34 (10.9%	0.434	9 (5.2%)	43 (12.5%)	0.009
Perineural invasion, *n* (%)	31 (15.1%)	44 (14.1%)	0.747	20 (11.5%)	55 (16.0%)	0.166
Liver capsule invasion, *n* (%)	134 (65.4%)	185 (59.3%)	0.165	123 (70.7%)	196 (57.1%)	0.003
Tumor differentiation, poor, *n* (%)	38 (19.5%)	42 (14.4%)	0.325	20 (11.8%)	60 (18.9%)	0.098
CA19-9, ≥22 U/mL, *n* (%)	50 (25.0%)	63 (20.6%)	0.244	142 (83.5%)	251 (74.7%)	0.054
HBsAg (positive), *n* (%)	77 (37.7%)	73 (23.5%)	<0.001	48(27.6%)	102 (29.9%)	0.583
HCV, *n* (%)	2 (1.0%)	2 (0.6%)	0.738	1 (0.6%)	2 (0.6%)	0.774
Child score, *n* (%)			0.306			0.001
5	170 (82.9%)	269 (86.2%)		160 (92.0%)	279 (81.3%)	
6	35 (17.1%)	43 (13.8%)		14 (8.0%)	64 (18.7%)	
TNM stage, III, *n* (%)	149 (72.6%)	212 (78%)	0.103	131 (75.3%)	230 (67.1%)	<0.001
Overall survival, month, median (interquartile range)	15.7 (7.9–26.7)	18.8 (11.9–37.7)	<0.001	19.2 (12.8–35.3)	16.7 (9.1–32.8)	0.046

STG, serum triglyceride; AGS, albumin-globulin score; CA19-9, cancer antigen 19-9; HBsAg, hepatitis B surface antigen; HCV, hepatitis C virus.

**TABLE 3:** Multivariate analyses to determine independent predictors of overall survival and recurrence-free survival in the discovery set.

Variables	Overall survival			Recurrence-free survival		
	HR	95% CI	*P*-value	HR	95% CI	*P*-value
Gender, female/male	0.89	0.70–1.15	0.4026	1.01	0.81–1.26	0.9562
Age, ≥60/<60 (years)	1.10	0.86–1.4	0.4402	1.03	0.84–1.29	0.7327
Impaired fasting glucose, ≥7/<7 (mmol/L)	1.42	0.89–1.97	0.0452	1.23	0.97–1.61	0.0997
Hepatolithiasis	1.11	0.82–1.53	0.4890	0.87	0.64–1.17	0.3567
Tumor number, Multiple/single	1.61	1.24–2.09	0.0003	1.62	1.28–2.05	<0.0001
Tumor size, ≥5/<5 (cm)	1.11	0.84–1.44	0.4652	1.22	0.96–1.56	0.1063
**Tumor differentiation**						
Well	Reference			Reference		
Moderate	1.30	0.73–2.34	0.3733	1.74	1.01–2.98	0.0463
Poor	2.26	1.21–4.26	0.0110	2.15	1.19–3.87	0.0110
Microvascular invasion	1.18	0.82–1.70	0.3746	1.42	1.02–1.99	0.0389
Node positivity	1.70	1.28–2.25	0.0002	1.38	1.06–1.79	0.0151
Liver capsule invasion	0.93	0.71–1.21	0.5752	0.96	0.76–1.23	0.7842
Perineural invasion	1.42	0.99–2.02	0.0501	1.24	0.89–1.72	0.1981
STG, ≥1/<1 (mmol/L)	0.66	0.52–0.85	0.0014	0.69	0.55–0.87	0.0014
**CAS**						
Grade 1	Reference			Reference		
Grade 2	1.54	1.14–2.06	0.0044	1.40	1.08–1.81	0.0100
Grade 3	2.84	1.90–4.24	<0.0001	2.29	1.58–3.33	<0.0001
CA199 grade, ≥22/<22 (U/ml)	1.92	1.44–2.57	<0.0001	1.49	1.14–1.95	0.0035

STG, serum triglyceride; CA19-9, cancer antigen 19-9; CAS, combination of albumin-globulin score and skeletal muscle index.

### Albumin-globulin score was associated with prognosis of intrahepatic cholangiocarcinoma patients after curative resection

Intrahepatic cholangiocarcinoma Patients were stratified into two groups according to the AGS; higher AGS stratification (1, 2) was associated with poor OS and RFS than the lower AGS stratification (0) in the discovery set (Figures 1C,D). In the validation set, marginal significance was likewise obtained on the difference between the two groups (*P* = 0.132) (Figure 2B). The correlations of clinicopathological characteristics with the AGS in the discovery and validation sets were exhibited in Table 2 and Supplementary Table 1.

### The proposal and validation of triglyceride-albumin-globulin grade

**FIGURE 3:**
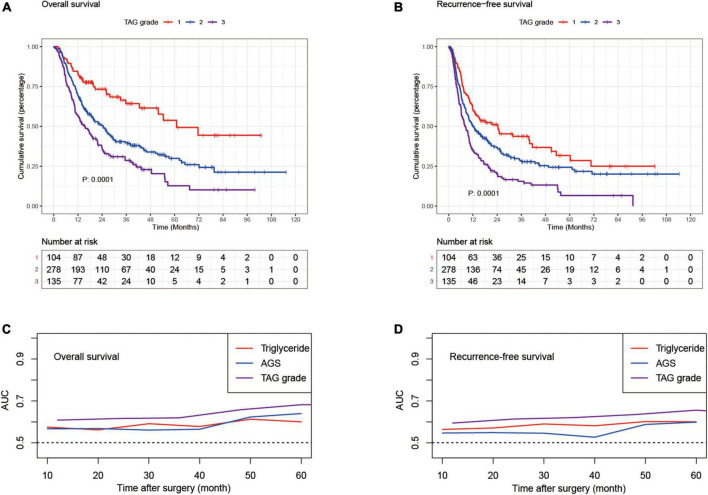
Kaplan–Meier curves for overall survival and recurrence-free survival stratified by TAG grade **(A,B)** in the discovery set; time-dependent AUROC curves of triglyceride, AGS and TAG grade for overall survival **(C)** and recurrence-free survival **(D)** in the discovery set; AUROC, area under receiver operating characteristic; AGS, albumin-globulin score; TAG grade, triglyceride-albumin-globulin grade.

**FIGURE 4:**
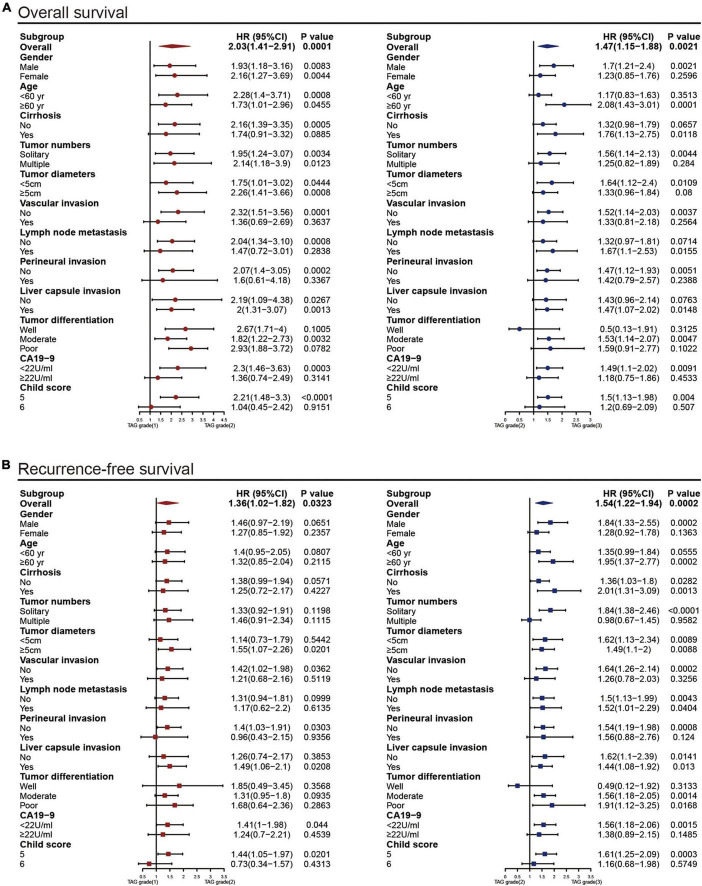
Subgroup analyses to assess the discrimination ability of the TAG grade for overall survival **(A)** and recurrence-free survival **(B)** in patients with different clinical characteristics; HR, hazard ratio; CI, confidence interval; TAG grade, triglyceride-albumin-globulin grade.

The triglyceride-albumin-globulin (TAG) grade was proposed as follows: patients with high TG level (≥1 mmol/L) and low AGS (0) were classified into TAG grade 1, those with low TG level (<1 mmol/L) and high AGS (1, 2) were classified into TAG grade 3, the rest of the patients were classified into TAG grade 2. In the discovery set, the patients in TAG grade 2 had superior 1-, 3-, and 5-year cumulative OS and RFS than those in TAG grade 3 (OS: 69.4, 24.1, and 8.6% vs. 57, 17.7, and 3.7%; RFS: 48.9, 16.2, and 6.8% vs. 34.1, 10.4, and 2.2%), but worse than those in TAG grade 1 (OS: 69.4, 24.1, and 8.6% vs. 83.7, 28.8, and 11.5%; RFS: 48.9, 16.2, and 6.8% vs. 60.6, 24, and 9.6%) (Figures 3A,B). A similar result was also generated in the patients of the validation set (Figure 2C). Moreover, the TAG grade possessed stronger predictive ability than the single use of TG or AGS through the result of time-dependent AUC analysis (Figures 3C,D). Subgroup analyses were conducted to further validate the efficacy of TAG grade in multiple clinical conditions, including different gender, age, number and diameter of tumor nodules, tumor differentiation, CA19-9 level and Child score; with or without liver cirrhosis, vascular invasion, positive lymph node, perineural and liver capsule invasion. The results of subgroup analyses shown in Figures 4A,B suggested that the ICC patients in higher TAG grades were related to decreased OS and RFS in various clinical conditions. Survival analyses based on Cox proportional hazard models in the discovery and validation sets presented that the TAG grade remained the independent risk factor for postoperative outcomes of surgically treated ICC patients (Table 4 and Supplementary Table 3). As shown in Figures 2D,E, the TAG grade demonstrated preferable net benefits within a wider range of threshold probability than the single use of either TG or AGS in predicting OS for the patients in both the discovery and validation sets. Moreover, in the validation set, the predicting abilities of the TAG grade were superior to the CAS grade in our previous study (21) (Figures 2F,G). The correlations between clinical characteristics and the TAG grade in the discovery and validation set were presented in Table 5 and Supplementary Table 2.

**TABLE 4:** Univariate and multivariate analyses to determine independent predictors of overall survival in the discovery set.

Variables	Univariate analysis			Multivariate analysis		
	HR	95% CI	*P*-value	HR	95% CI	*P*-value
Gender, female/male	0.8073	0.64–1	0.0606	0.828	0.64–1.06	0.1348
Age, ≥60/<60 (years)	0.9603	0.76–1.2	0.7239			
Cirrhosis	1.2573	0.98–1.6	0.066	1.6133	1.23–2.12	0.0006
Hepatolithiasis	1.3263	1.1–1.75	0.0484	1.1299	0.83–1.55	0.4465
Tumor number, multiple/single	1.6661	1.32–2.1	<0.0001	1.6544	1.27–2.15	0.0002
Tumor size, ≥5/<5 (cm)	1.2161	0.96–1.52	0.0916	1.1344	0.87–1.48	0.3523
**Tumor differentiation**						
Well	Reference			Reference		
Moderate	1.79	1.02–3.13	0.0415	1.188	0.67–2.12	0.5602
Poor	2.6893	1.46–4.92	0.0014	1.8792	1–3.53	0.0497
Microvascular invasion	1.7008	1.22–2.37	0.0018	1.1927	0.83–1.71	0.3411
Vascular invasion	1.1532	0.89–1.49	0.2816	0.9944	0.74–1.33	0.9699
Node positivity	2.3488	1.85–2.98	<0.0001	1.8416	1.39–2.43	<0.0001
Liver capsule invasion	1.0749	0.85–1.35	0.5392			
Perineural invasion	1.5583	1.15–2.11	0.004	1.3597	0.95–1.94	0.0892
**TAG grade**						
1	Reference			Reference		
2	2.0244	1.41–2.9	0.0001	1.9632	1.35–2.86	0.0004
3	2.9832	2.04–4.37	<0.0001	2.443	1.64–3.65	<0.0001
CA199 grade ≥22/<22 (U/ml)	2.5093	1.96–3.21	<0.0001	2.0296	1.52–2.72	<0.0001
HBV	1.1162	0.87–1.43	0.3783			

TAG grade, triglyceride-albumin-globulin grade; CA19-9, cancer antigen 19-9; HBV, hepatitis B virus.

**TABLE 5:** Comparison of characteristics with the different TAG grades of 517 ICC patients treated with surgical resection in the discovery set.

Characteristics	TAG grade			
	1 (*n* = 104)	2 (*n* = 278)	3 (*n* = 135)	*P*-value
Age, mean ± *SD*	57.7 ± 10.3	57.6 ± 10.9	56.1 ± 10.4	0.332
Gender, male, *n* (%)	54 (51.9%)	128 (46.0%)	69 (51.1%)	0.466
BMI kg/m^2^, mean ± *SD*	24.9 ± 4.8	26.1 ± 4.3	28.6 ± 5.9	0.635
Cirrhosis, *n* (%)	31 (29.8%)	68 (24.5%)	46 (34.1%)	0.113
Ascites, *n* (%)	10 (9.6%)	19 (6.8%)	19 (14.1%)	0.059
Multiple tumors, *n* (%)	30 (28.8%)	83 (29.9%)	43 (31.9%)	0.869
Tumor size (<5 cm), *n* (%)	45 (43.3%)	122 (43.9%)	58 (43.0%)	0.983
Hepatolithiasis, *n* (%)	16 (15.4%)	44 (15.8%)	27 (20.0%)	0.516
Microvascular invasion, *n* (%)	10 (9.6%)	23 (8.3%)	19 (14.1%)	0.182
Vascular invasion, *n* (%)	24 (23.1%)	60 (21.6%)	36 (26.7%)	0.517
Node positivity, *n* (%)	15 (14.4%)	70 (25.2%)	42 (31.1%)	0.011
Biliary invasion, *n* (%)	6 (5.8%)	31 (11.2%)	15 (11.1%)	0.266
Perineural invasion, *n* (%)	12 (11.5%)	40 (14.4%)	23 (17.0%)	0.487
Liver capsule invasion, *n* (%)	74 (71.2%)	160 (57.6%)	85 (63.0%)	0.049
Tumor differentiation, poor, *n* (%)	10 (9.9%)	42 (16.2%)	28 (22.0%)	0.176
CA19-9, ≥22 U/mL, *n* (%)	83 (81.4%)	219 (80.5%)	91 (68.9%)	0.019
HBsAg (positive), *n* (%)	22 (21.2%)	77 (27.8%)	51 (38.1%)	0.013
HCV, *n* (%)	0 (0.0%)	2 (0.7%)	1 (0.7%)	0.803
Child score, *n* (%)				0.008
5	94 (90.4%)	241 (86.7%)	104 (77.0%)	
6	10 (9.6%)	37 (13.3%)	31 (23.0%)	
TNM stage, III, *n* (%)	79 (76.0%)	185 (66.5%)	97 (71.8%)	0.056
Overall survival, month, median (interquartile range)	20.1 (14.0–38.1)	17.9 (10.7–33.5)	14.0 (7.2–27.4)	<0.001

TAG grade, triglyceride-albumin-globulin grade; CA19-9, cancer antigen 19-9; HBsAg, hepatitis B surface antigen; HCV, hepatitis C virus.

## Discussion

The present study revealed that elevated preoperative serum TG was an independent protective factor for ICC patients after curative liver resection. TG showed the greatest predictive effectiveness among several parameters related to hepatic function and serum lipid level. Likewise. AGS demonstrated discriminative capability in predicting long-term surgical outcomes of the ICC patients. The serum TG level and AGS were further combined into a novel nutrition grade called TAG grade, the ICC patients in different TAG grades displayed markedly different postoperative survival rates; these differences were maintained in various subgroups as well. Additionally, with superior predictive ability than the TG and AGS, the TAG grade was a desirable model in the prognosis prediction of ICC patients who underwent curative hepatectomy.

There is evolving evidence that preoperative serum markers play essential roles in the prognosis prediction of patients with primary liver cancers. Conventional markers including AFP, PIVKA-II, and carbohydrate antigen 19-9 (CA19-9) have been extensively studied and applied in HCC and ICC patients (26, 27). By combining inflammation-related indexes (counts of lymphocyte, monocyte, neutrophil and platelet; concentration of C-reactive protein) and liver function parameters [levels of bilirubin, albumin, aspartate aminotransferase (AST), alanine aminotransferase (ALT), alkaline phosphatase (ALP), and gamma-glutamyl transpeptidase (GGT)], several prognostic scores including the Glasgow Prognostic Score (GPS), Prognostic Nutritional Index (PNI), and lymphocyte-C-reactive protein ratio (LCR) were introduced and validated in predicting outcomes of ICC patients following surgical treatments (28, 29). Nutritional status has been broadly reported to be implicated in cancer progression and prognosis (30); an effective nutritional assessment before surgery is crucial for achieving optimal short- and long-term survival of ICC patients. In our previous study (21), the SMI generated from the CT was used to evaluate the nutritional status of ICC patients prior to the operation, which exhibited a good clinical efficacy. However, interpretation of the radiographic based SMI required the assistances of the experienced radiologists, which limited its application, especially in primary health-care facilities. therefore, an easy-to-use serological index was needed. Notwithstanding, serological indicators for preoperative evaluation of nutritional status are limited.

TG is the most common type of fat derived from dietary intake or extra calories. The correlation between circulating TG concentration and a range of metabolic diseases including diabetes and CHD has been extensively studied recently (31, 32). In 1991, elevated serum TG level was reported to be associated with a higher incidence of developing breast cancer (33). In the male population, fasting TG and glucose levels were significantly correlated with the risk of non-small-cell lung cancer (34). In a large cohort study involving 1,56,153 participants in Austria, serum TG concentration was found to be positively or reversely associated with multiple malignancies (13). Liu et al. (14) demonstrated that the serum TG level less than 0.81 mmol/L was a predictor for a worse prognosis of HCC patients in the absence of liver cirrhosis. Andreotti et al. (15) reported a positive correlation between STG and biliary malignancies (gall bladder, extrahepatic bile duct and the ampulla of Vater). Interestingly, a robust negative correlation between TG level and poor prognosis of ICC patients following liver resection was discovered in the present study. Apart from conventional risk factors such as primary sclerosing cholangitis, biliary tract cysts and hepatolithiasis for both ECC and ICC; chronic hepatitis, obesity, alcoholic, and non-alcoholic liver diseases are emerging as major concerns in the etiology of ICC (35). These diversities may explain why the increased STG levels were associated with higher prevalence of ECC but better prognosis of ICC.

The mechanism underlying the relationship between TG and liver cancer has not been fully elucidated. Lipid metabolism is emerging as an essential factor in tumor initiation and progression; several targets evolved in lipid metabolism have been reported as potential therapeutic targets in the treatment of primary liver cancer. During the *De- novo* FA synthesis of liver cancer, glucose is taken up in the HCC cell and converted into FA for storage in the form of TG, which plays crucial roles in cancer cell survival by inducing autophagy, influencing intracellular signaling and gene expression, meanwhile increasing energy production (36). Related targets include SCD1 (37), FASN (38) and ACC (39) and their inhibitors have been intensely investigated in the proliferation and metastasis of liver cancer. Moreover, some of the canonical cancer signal transduction pathways such as AMP-activated protein kinase (AMPK) (40), Wnt and Ras pathways were considered to have an impact on TG composition, thus affecting hepatic tumorigenesis (41).

The ALB is one of the most frequently used markers for evaluating the nutritional statuses of cancer patients (42). Serum ALB may act as a tumor suppressor through the following mechanisms: on the one hand, the activated pro-inflammatory cytokines including IL-6 and TNF-α inhibit the secretion of ALB by hepatic cells, these cytokines are critical molecules driving liver cancer progression (43); on the other hand, ALB serves as a stabilizer of cell growth and DNA replication by scavenging the free radicals, thereby maintains the endocrine homeostasis (44).

Given the prognostic values of TG and AGS in surgically treated ICC patients, a novel nutritional model (TAG grade) was proposed by combining these two markers. The TAG grade exhibited optimal discriminatory capability and reliable clinical efficacy, superior to the CT-based CMI grade in our previous study (21).

This study is noticeable in the following aspects. Firstly, this was the first study to elucidate the correlation between serum TG and prognosis of ICC in a large cohort of 631 patients including multiple medical centers. Secondly, as far as we are aware, this was the first study that combined serum TG, ALB, and GLB levels to assess the long-term survival of ICC patients after curative resection. The results may be profitable for nutritional assessment and patient selection prior to surgical treatment.

Despite the positive outcomes, there were significant restrictions: (1) the two involved centers were situated in the southwest part of mainland China, because of the high frequency of hepatolithiasis in this area, the accuracy of the TAG grade may be restricted by the etiological agents and retrospective design, further prospective worldwide studies were required to confirm our initial findings. (2) A small part of patients underwent retreatment at different medical facilities following tumor recurrence; as a result, this study was unable to examine the impact of postoperative treatment for recurrent ICC, which may affect the outcomes of this part of patients. (3) The glucose level was not assessed as a covariate; given the potential impact that the hyperglycemia had on the TG level, the results might be confounded.

## Conclusion

Elevated serum TG concentration was independently associated with better long-term outcomes of ICC patients following curative hepatectomies. It is beneficial to consider the proposed TAG grade as a surrogate nutritional score in risk classification for surgically treated ICC patients.

## Data availability statement

The raw data supporting the conclusions of this article will be made available by the authors, without undue reservation, to any qualified researcher.

## Ethics statement

Written informed consents were obtained from all participants or their entrusted agents. This study was approved by the Ethics Committee of West China Hospital of Sichuan University and the First Affiliated Hospital of Chongqing Medical University, following the guidelines of the 1975 Declaration of Helsinki.

## Author contributions

YC, TL, and HW: conceptualization. YC, JL, HX, and SX: data curation. YC and TL: formal analysis and writing—original draft. HW and TL: supervision. All authors contributed to the article and approved the submitted version.

## Footnotes

1
http://www.empowerstats.com

2
https://www.r-project.org

3
http://medicine.yale.edu/lab/rimm/research/software

## Abbreviations

ACC: acetyl CoA carboxylase
AFP: alpha-fetoprotein
AGS: albumin-globulin score
AJCC: American Joint Committee on Cancer
ALB: albumin
ALBI: albumin-bilirubin index
ALD: alcoholic liver disease
ALP: alkaline phosphatase
ALT: alanine aminotransferase
AMPK: AMP-activated protein kinase
AST: aspartate aminotransferase
AUC: area under curve
BMI: body mass index
CAS: combination of albumin-globulin score and skeletal muscle index
CA19-9: cancer antigen 19-9
CEA: carcinoembryonic antigen
CHD: coronary heart disease
CHOL: cholesterol
CT: computed tomography
DCA: decision curve analysis
DNA: deoxyribonucleic acid
ECC: extrahepatic cholangiocarcinoma
FA: fatty acid
FASN: fatty acid synthase
FIB-4: fibrosis-4 index
GLB: globulin
GPS: glasgow prognostic score
HBV: hepatitis B virus
HCC: hepatocellular carcinoma
HCV: hepatitis C virus
ICC: intrahepatic cholangiocarcinoma
IL-6: interleukin-6
INR: international normalized ratio
LCR: lymphocyte-C-reactive protein ratio
LDL-C: low-density lipoprotein
MVI: microvascular invasion
NAFLD: non-alcoholic fatty liver disease
NLR: neutrophil-to-lymphocyte ratio
OS: overall survival
PIVKA-II: prothrombin induced by vitamin K absence-II
PNI: prognostic nutritional index
RFA: radiofrequency ablation
RFS: recurrence-free survival
ROC: receiver operating characteristic
SCD-1: stearoyl-CoA desaturase-1
SMI: skeletal muscle index
STG: serum triglyceride
TACE: transarterial chemoembolization
TAG grade: triglyceride-albumin-globulin grade
TNF-α: tumor necrosis factor-α
TNM: tumor-node-metastasis.
