# Supplementary material for: A novel nutritional score based on serum triglyceride and protein levels predicts outcomes of intrahepatic cholangiocarcinoma after curative hepatectomy: A multi-center study of 631 patients
Source: Front Nutr. 2022 Sep 21;9:964591. doi: 10.3389/fnut.2022.964591 (PMC9533229; doi:10.3389/fnut.2022.964591)
Supplement: Supplementary file 1 [file Data_Sheet_1.pdf]

Supplementary tables

Supplementary table 1. Comparison of characteristics with the different serum triglyceride levels and AGS grades of 114 ICC patients treated with surgical resection in the validation set.

Characteristics	STG (mmol/L)		P value	AGS		P value
	<1 (n=53)	≥1 (n=61)		Low (0) (n=28)	High (1, 2) (n=86)	
Age, mean ± SD	55.7 ± 12.0	60.5 ± 10.1	0.054	55.8 ± 10.5	59.1 ± 11.3	0.165
Gender, male, n (%)	26 (49.1%)	23 (37.7%)	0.222	12 (42.9%)	37 (43.0%)	0.988
BMI kg/m^2, mean ± SD	25.8 ± 5.3	23.3 ± 2.9	0.121	24.4 ± 3.3	26.8 ± 5.6	0.898
Cirrhosis, n (%)	10 (18.9%)	6 (9.8%)	0.166	4 (14.3%)	12 (14.0%)	0.965
Ascites, n (%)	24 (45.3%)	31 (50.8%)	0.555	14 (50.0%)	41 (47.7%)	0.831
Multiple tumors, n (%)	16 (30.2%)	16 (26.2%)	0.639	6 (21.4%)	26 (30.2%)	0.368
Tumor size (<5 cm), n (%)	19 (35.8%)	27 (44.3%)	0.361	11 (39.3%)	35 (40.7%)	0.895
Hepatolithiasis, n (%)	8 (15.1%)	13 (21.3%)	0.393	5 (17.9%)	16 (18.6%)	0.929
Microvascular invasion, n (%)	36 (67.9%)	34 (55.7%)	0.182	13 (46.4%)	57 (66.3%)	0.061
Vascular invasion, n (%)	36 (67.9%)	35 (57.4%)	0.247	14 (50.0%)	57 (66.3%)	0.123
Node positivity, n (%)	15 (28.3%)	16 (26.2%)	0.804	6 (21.4%)	25 (29.1%)	0.43
Biliary invasion, n (%)	27 (50.9%)	31 (50.8%)	0.989	11 (39.3%)	47 (54.7%)	0.158
Perineural invasion, n (%)	12 (22.6%)	13 (21.3%)	0.864	6 (21.4%)	19 (22.1%)	0.941
Liver capsule invasion, n (%)	33 (62.3%)	29 (47.5%)	0.115	16 (57.1%)	46 (53.5%)	0.736
Tumor differentiation, poor, n (%)	41 (77.4%)	43 (70.5%)	0.406	19 (67.9%)	65 (75.6%)	0.42
CA19-9, ≥22U/mL, n (%)	41 (77.4%)	44 (72.1%)	0.523	16 (57.1%)	69 (80.2%)	0.015
HBsAg (positive), n (%)	15 (28.3%)	6 (9.8%)	0.011	3 (10.7%)	18 (20.9%)	0.226
HCV, n (%)	1 (1.9%)	0 (0.0%)	0.465	0 (0.0%)	1 (1.2%)	1
Child score, n (%)			0.994			<0.001
5	40 (75.5%)	46 (75.4%)		28 (100.0%)	58 (67.4%)	
6	13 (24.5%)	15 (24.6%)		0 (0.0%)	28 (32.6%)	
TNM stage, III, n (%)	35 (66.0%)	36 (59.0%)	0.44	16 (57.1%)	55 (64.0%)	0.518
Overall survival, month, median (interquartile range)	17.1 (6.7-56.9)	30.9 (9.8-61.1)	0.076	53.2 (9.6-65.2)	21.8 (9.2-54.3)	0.264

Abbreviations: STG, serum triglyceride; AGS, albumin-globulin score; CA19-9, cancer antigen 19-9; HBsAg, hepatitis B surface antigen; HCV, hepatitis C virus.

Supplementary table 2. Comparison of characteristics with the different TAG grades of 114 ICC patients treated with surgical resection in the validation set.

Characteristics	TAG grade			P value
	1 (n=17)	2 (n=55)	3 (n=42)	
Age, mean ± SD	57.8 ± 10.4	59.8 ± 10.5	56.5 ± 12.3	0.436
Gender, male, n (%)	5 (29.4%)	25 (45.5%)	19 (45.2%)	0.472
BMI kg/m^2, mean ± SD	22.7 ± 3.6	23.1 ± 2.9	25.8 ± 3.1	0.326
Cirrhosis, n (%)	1 (5.9%)	8 (14.5%)	7 (16.7%)	0.552
Ascites, n (%)	10 (58.8%)	25 (45.5%)	20 (47.6%)	0.625
Multiple tumors, n (%)	3 (17.6%)	16 (29.1%)	13 (31.0%)	0.572
Tumor size (<5 cm), n (%)	7 (41.2%)	24 (43.6%)	15 (35.7%)	0.731
Hepatolithiasis, n (%)	4 (23.5%)	10 (18.2%)	7 (16.7%)	0.826
Microvascular invasion, n (%)	8 (47.1%)	31 (56.4%)	31 (73.8%)	0.091
Vascular invasion, n (%)	8 (47.1%)	33 (60.0%)	30 (71.4%)	0.193
Node positivity, n (%)	4 (23.5%)	14 (25.5%)	13 (31.0%)	0.779
Biliary invasion, n (%)	6 (35.3%)	30 (54.5%)	22 (52.4%)	0.37
Perineural invasion, n (%)	4 (23.5%)	11 (20.0%)	10 (23.8%)	0.891
Liver capsule invasion, n (%)	8 (47.1%)	29 (52.7%)	25 (59.5%)	0.645
Tumor differentiation, poor, n (%)	12 (70.6%)	38 (69.1%)	34 (81.0%)	0.401
CA19-9, ≥22U/mL, n (%)	9 (52.9%)	42 (76.4%)	34 (81.0%)	0.075
HBsAg (positive), n (%)	0 (0.0%)	9 (16.4%)	12 (28.6%)	0.032
HCV, n (%)	0 (0.0%)	0 (0.0%)	1 (2.4%)	0.518
Child score, n (%)				0.035
5	17 (100.0%)	40 (72.7%)	29 (69.0%)	
6	0 (0.0%)	15 (27.3%)	13 (31.0%)	
TNM stage, III, n (%)	9 (52.9%)	34 (61.8%)	28 (66.7%)	0.613
Overall survival, month, median (interquartile range)	52.2 (9.6-59.2)	26.8 (11.4-64.7)	16.5 (6.9-43.1)	0.087

Abbreviations: TAG grade, triglyceride-albumin-globulin grade; CA19-9, cancer antigen 19-9; HBV, hepatitis B virus.

Supplementary table 3. Univariate and multivariate analyses to determine independent predictors of overall survival in the validation set.

Variables	Univariate analysis			Multivariate analysis		
	HR	95%CI	P value	HR	95%CI	P value
Gender, Female/Male	0.982	0.62-1.56	0.9388			
Age, ⩾60/<60(years)	0.991	0.62-1.57	0.9695			
Cirrhosis	1.3955	0.78-2.5	0.2639			
Hepatolithiasis	1.7803	1.03-3.07	0.038	2.5792	1.27-5.23	0.0086
Tumor number, Multiple/single	1.4726	0.9-2.4	0.1212	1.3654	0.79-2.37	0.268
Tumor size,⩾ 5/<5(cm)	1.5982	0.99-2.58	0.0558	1.7403	1.03-2.95	0.0397
Tumor differentiation						
Well	Reference			Reference		
Moderate	1.8447	1.16-3.15	0.0445	1.7549	1.03-3.79	0.0562
Poor	2.2669	1.24-4.14	0.0077	2.1126	1.05-4.26	0.0363
Microvascular invasion	2.2208	0.87-5.61	<0.0001	2.456	0.92-4.37	0.0515
Vascular invasion	3.1063	1.13-5.66	<0.0001	2.9743	0.96-4.33	0.0003
Node positivity	2.8774	1.77-4.67	<0.0001	1.909	1.12-3.25	0.017
Liver capsule invasion	1.46	0.91-2.33	0.1138	1.14	0.79-2.21	0.078
Perineural invasion	2.2919	1.37-3.83	0.0015	1.3654	0.78-2.39	0.2747
TAG grade						
1	Reference			Reference		
2	1.3261	0.61-2.87	0.4744	1.3112	0.55-3.13	0.0241
3	2.1712	0.99-4.73	0.0509	2.097	0.86-5.1	0.0041
CA199 grade, ⩾22/<22(U/ml)	3.5858	1.78-7.23	0.0004	1.6635	0.78-3.53	0.185
HBV	0.8607	0.47-1.57	0.6243			

Abbreviations: TAG grade, triglyceride-albumin-globulin grade; CA19-9, cancer antigen 19-9; HBV, hepatitis B virus.

Supplementary figure legends

Supplementary figure 1. Identification for the cutoff values of TG (**A**), CHOL (**B**) and LDL-C (**C**) by X-tile analyses in the discovery set; TG, triglyceride; CHOL, cholesterol; LDL-C, low-density lipoprotein.

Supplementary figure 2. Kaplan-Meier curves for overall survival (**A**) and recurrence-free survival (**B**) stratified by the upper limit of normal range of the triglyceride concentration in the discovery set.

Supplementary figure 3. The ROC curves analyses to compare the ability of TG, FIB-4 and ALBI in predicting 1-year (**A, B**), 3-year (**C, D**) and 5-year (**E, F**) overall and recurrence-

free survival in the discovery set; TG, triglyceride; FIB-4, fibrosis-4 index; ALBI, albumin-bilirubin index.
