# Supplementary figures and images for: A novel nutritional score based on serum triglyceride and protein levels predicts outcomes of intrahepatic cholangiocarcinoma after curative hepatectomy: A multi-center study of 631 patients
Source: Front Nutr. 2022 Sep 21;9:964591. doi: 10.3389/fnut.2022.964591 (PMC9533229; doi:10.3389/fnut.2022.964591)

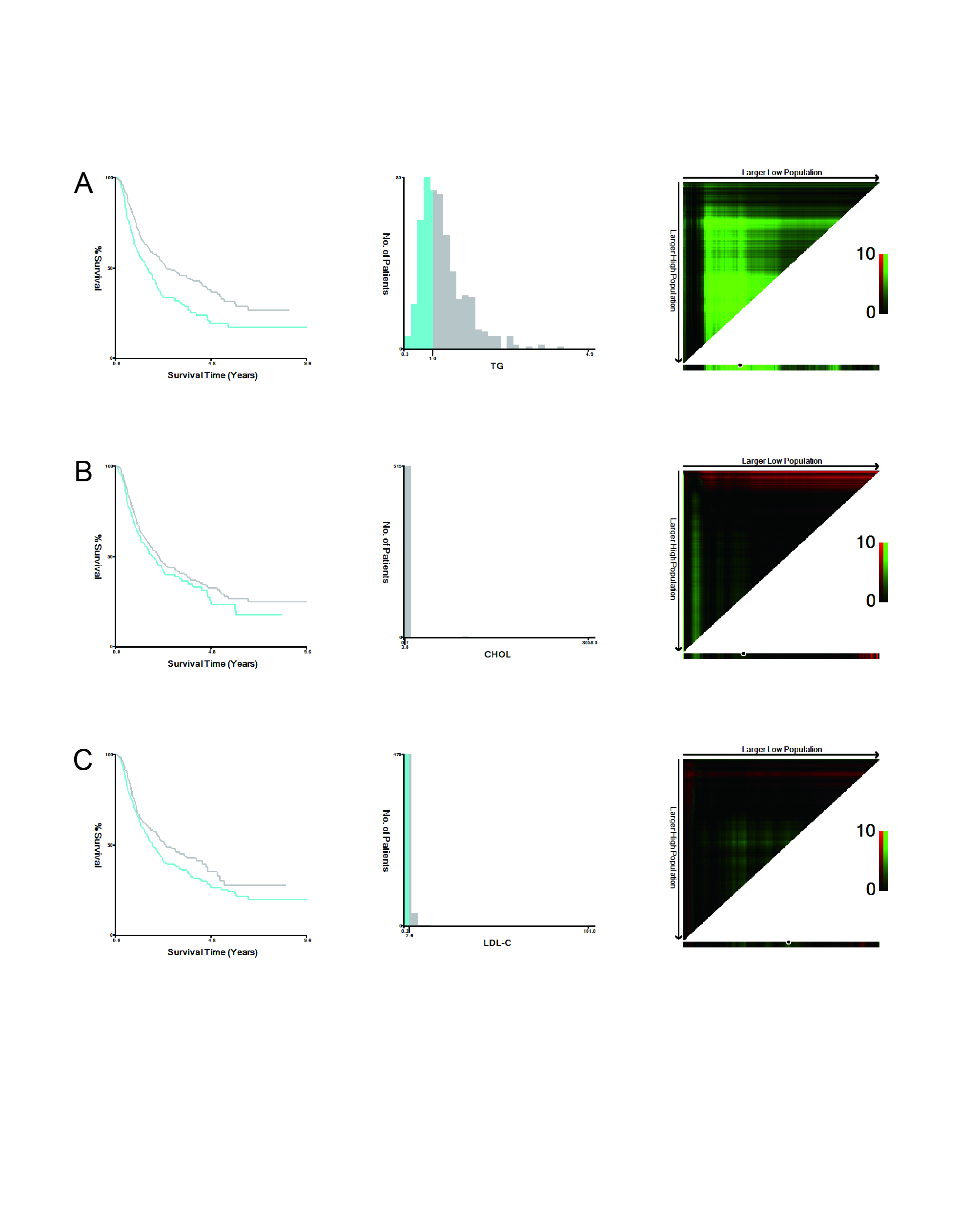

Supplement: Supplementary file 2 [file Image_1.TIF]

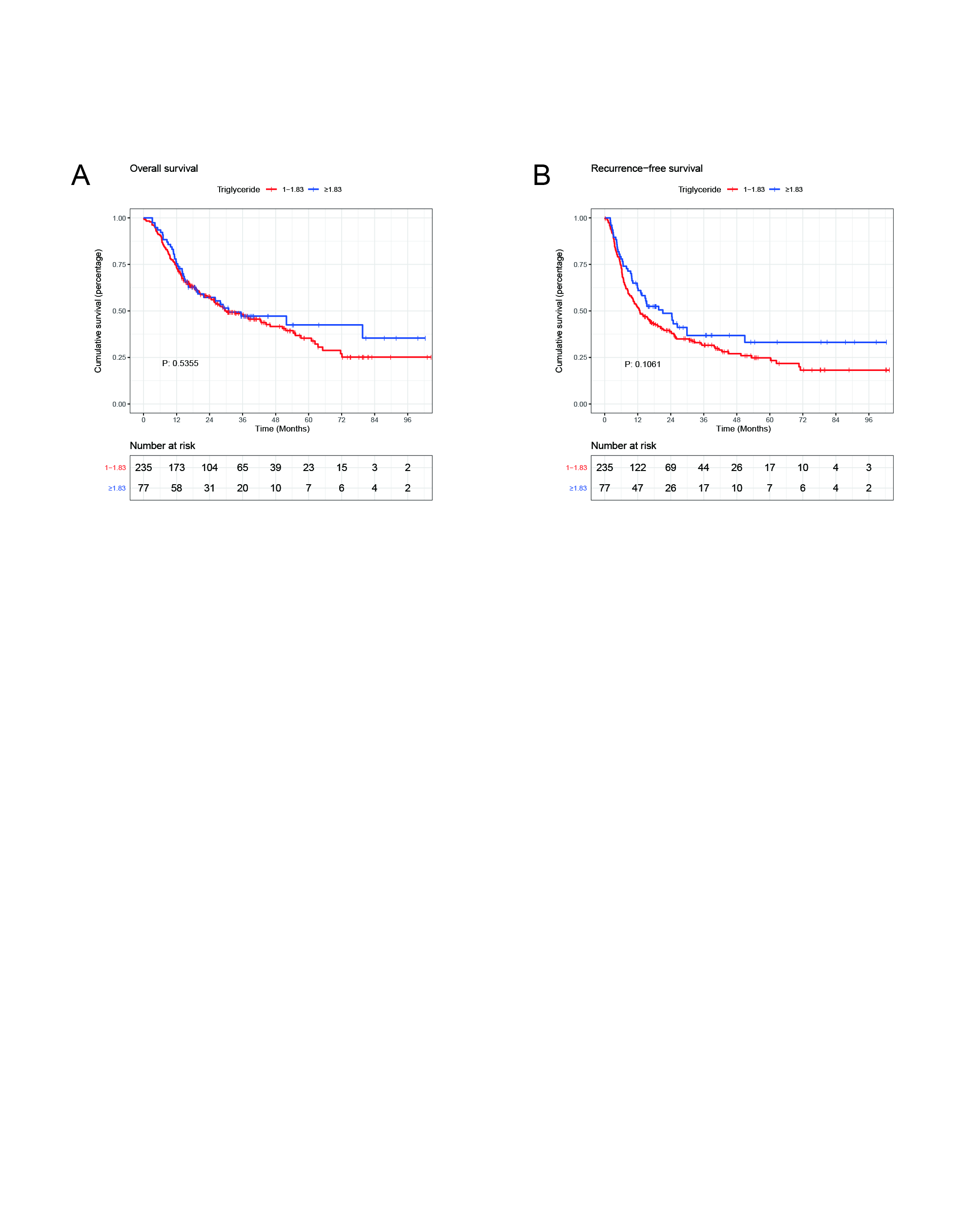

Supplement: Supplementary file 3 [file Image_2.TIF]

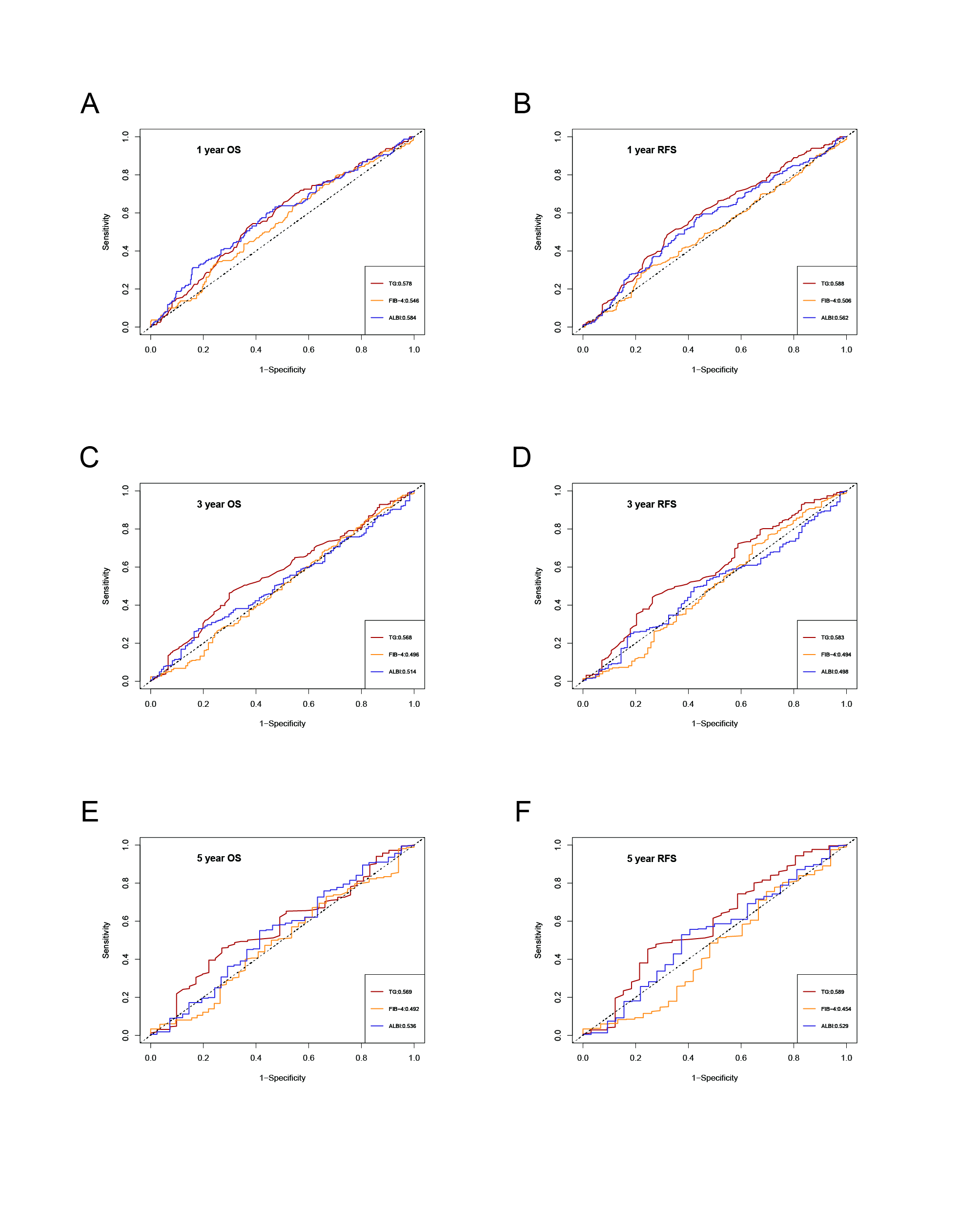

Supplement: Supplementary file 4 [file Image_3.TIF]
